# Flavonoids as Important Molecules of Plant Interactions with the Environment

**DOI:** 10.3390/molecules191016240

**Published:** 2014-10-10

**Authors:** Justyna Mierziak, Kamil Kostyn, Anna Kulma

**Affiliations:** Faculty of Biotechnology, Wroclaw University, Przybyszewskiego 63/77, 51-148 Wroclaw, Poland

**Keywords:** secondary metabolites, flavonoids, antioxidants, plant protection

## Abstract

Flavonoids are small molecular secondary metabolites synthesized by plants with various biological activities. Due to their physical and biochemical properties, they are capable of participating in plants’ interactions with other organisms (microorganisms, animals and other plants) and their reactions to environmental stresses. The majority of their functions result from their strong antioxidative properties. Although an increasing number of studies focus on the application of flavonoids in medicine or the food industry, their relevance for the plants themselves also deserves extensive investigations. This review summarizes the current knowledge on the functions of flavonoids in the physiology of plants and their relations with the environment.

## 1. Introduction

**Figure 1:**
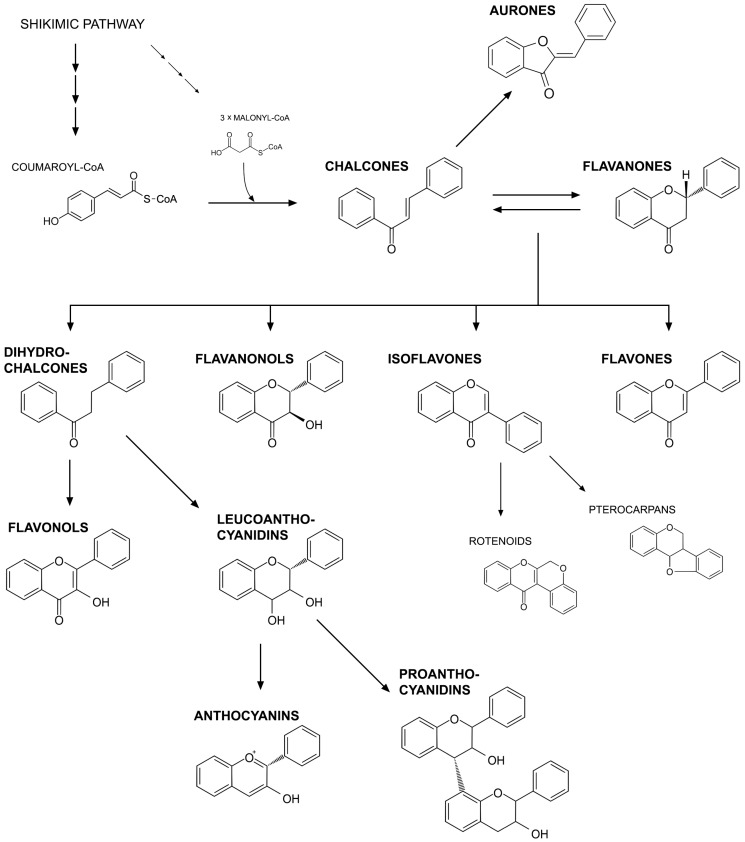
Classification of flavonoids.

Flavonoids are plant secondary metabolites, derivatives of 2-phenyl-benzyl-γ-pyrone, present ubiquitously throughout the plant kingdom. Over 9,000 compounds of this group are known [1]. Their biosynthesis pathway (part of the phenylpropanoid pathway) begins with the condensation of one p-coumaroyl-CoA molecule with three molecules of malonyl-CoA to yield chalcone (4',2',4',6'-tetrahydroxychalcone), catalyzed by chalcone synthase (CHS). The next step is isomerization of chalcone to flavanone by chalcone isomerase (CHI). From this step onwards, the pathway branches to several different flavonoid classes, including aurones, dihydrochalcones, flavanonols (dihydroflavonols), isoflavones, flavones, flavonols, leucoanthocyanidins, anthocyanins and proanthocyanidins (Figure 1). Flavonoids undergo further modifications, for example methylation by methyltransferases and glycosylation by specific glycosyltransferases. These modifications often alter their solubility, reactivity and stability. The majority of flavonoids are present in the form of glycosides under natural conditions [2,3]. Because of their diverse chemical structure and variety resulting from the attached substituents, they have a number of important functions in plants. They participate in plant protection against biotic (herbivores, pathogens) and abiotic stresses (UV radiation, heat), and due to their antioxidative properties, they also maintain a redox state in cells. The antioxidative activity of flavonoids is connected with the structure of the molecule: the presence of conjugated double bonds and the occurrence of functional groups in the rings [4,5,6]. Flavonoids reduce the production of and quench reactive oxygen species (ROS) through:

1. suppression of singlet oxygen;
2. inhibition of enzymes that generate ROS (cyclooxygenase, lipoxygenase, monooxygenase, xanthine oxidase);
3. chelating ions of transition metals, which may catalyze ROS production;
4. quenching cascades of free-radical reactions in lipid peroxidation;
5. “re-cycling” of other antioxidants [4,7,8,9,10,11].

Due to their low redox potential, they can reduce strong free radicals (superoxides, alkyl radicals, hydroxyl radicals) [12].

## 2. Flavonoids in the Relations between Plants and Animals

Flavonoids are synthesized in all parts of the plant. They play a role in providing color, fragrance and taste to the fruits, flowers and seeds, which makes them attractants for insects, birds or mammals, which aid in pollen or seed transmission [13]. Plants release various chemicals both to deter and attract insects, in some cases natural predators of herbivores feeding on a plant. Flavonoids are among the chemicals that have been reported to regulate oviposition and feeding. Naringenin, hesperetin-7-*O*-rutinoside and quercetin-3-*O*-rutinoside, along with other active compounds, stimulated oviposition in swallowtail butterfly *Papilio* on young leaves of citrus plants [14]. Similar activity was found for luteolin 7-*O*-(6''-malonyl glucoside) on *Papilio polyxenes* [15] and for isorhamnetin glucoside on *Luehdorfia japonica* oviposition on the leaves of plants of the *Asarum* genus [16]. Flavonoids can also prevent insects from laying eggs, e.g., quercetin-3-*O*-rutinoside acts as a stimulant to *Danaus plexippus*, but as a deterrent to *Pieris rapae* [17,18].

It has been reported that the maize response to corn earworm, *Helicoverpa zea*, is mainly due to the presence of the C-glycosyl flavone, maysin (2"-*O*-a-l-rhamnosyl-6-C-(6-deoxy-xylo-hexos-4-ulosyl) luteolin), and the phenylpropanoid, chlorogenic acid [19]. Flavonoids, such as flavones 5-hydroxyisoderricin, 7-methoxy-8- (3-methylbutadienyl)-flavanone and 5-methoxyisoronchocarpin, and isoflavonoids (judaicin, judaicin-7-*O*-glucoside, 2-methoxyjudaicin and maackiain), were also reported as direct feeding deterrents. Flavonoids are cytotoxic and interact with different enzymes through complexation. Both flavonoids and isoflavonoids protect the plant against insect pests by influencing their behavior, growth and development [20,21].

Flavonoids play an important role in the protection of plants against plant feeding insects and herbivores [8]. Their presence can alter the palatability of the plants and reduce their nutritive value, decrease digestibility or even act as toxins. A study on a mixture of flavonoids from *Cistus ladanifer* L. that contained apigenin and 3,7-di-*O*-methylkaempferol demonstrated that they can influence calcium-dependent ATPase in the skeletal muscle sarcoplasmic reticulum and lead to deterioration of muscle relaxation [22]. A number of insect species have been shown to be sensitive to flavonoid compounds in feeding tests [23]. Rutin and quercetin-3-glucoside contained in *Pinus banksiana* inhibit the development and increase the mortality of *Lymantria dispar* [24]. Studies on peanuts revealed that the amounts of quercetin and rutin glycosides are related to increased mortality of the tobacco armyworm (*Spodoptera litura*) [25]. In rice, three flavone glucosides that inhibit digestion in insects and function as deterrent agents towards *Nilaparvata lugens* were identified [8]. Isoflavonoids and proanthocyanidins are other classes of flavonoids responsible for plant protection against insects. For example, naringenin procyanidin inhibits the development of *Aphis craccivora* [8].

Flavonoids can also have deterrent properties, with respect to feeding and physiological behavior, against soil nematodes, which feed on plants. For instance, kaempferol, quercetin and myricetin (flavonols) act as deterrents against *Radopholus similis* and *Meloidogyne incognita*, while genistein and daidzein (isoflavones) are active against *Radopholus similis*. The flavonols inhibit peristalsis of some nematodes and kaempferol, in addition to restricting their hatching [26].

Although flavonoid compounds may act as attractants or feeding/growth stimulators for certain insect species, they are of high relevance for the plant defense mechanism.

## 3. Flavonoids as Regulators of Symbiotic Interactions with Microorganisms

Flavonoids can act as specific transmitters in symbiotic relations between species, in particular, symbiotic bacteria. Low soil nitrogen concentration induces accumulation of flavonoids, which act as attractants for diazotrophs, resulting in the transport of reduced nitrogen forms to plant cells, while the bacteria utilize the plant’s photosynthesis products.

Naringenin can stimulate the colonization of wheat roots by *Azorhizobium caulinodans* [27]. Flavonoids, such as luteolin and chrysin, excreted by legumes, e.g., *Medicago sativa*, act as a specific signal for *Rhizobium* bacteria to initiate symbiosis [28]. The release is strongest at root tips and the emerging root hair zone, which are the target sites for *Rhizobium* infections [29].

**Figure 2:**
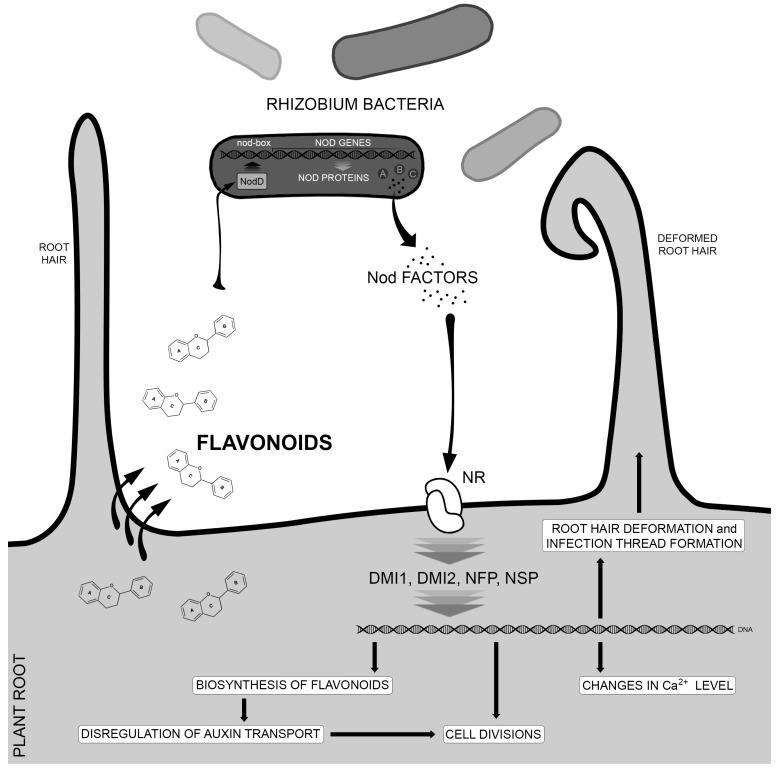
The role of flavonoids in the interactions between nitrogen-fixing bacteria and legume plants. Flavonoids are released by the plant bind to and activate NodD protein, which, in turn, attaches to no-box cassettes in the promoter sequences of nod genes, leading to the production of nod proteins and subsequently nod factors. These molecules bind to the nod factor receptors (NR) localized on the plant cell membrane, starting a cascade of signal transduction. This signal activates various gene expression, leading to root hair deformation, formation of infection thread, flavonoid biosynthesis, cell division and, finally, root nodule formation.

Flavonoids can influence, both positively and negatively, the expression level of bacterial *nod* genes, which control root nodule formation in the nitrogen-fixing bacteria [30,31,32]. For instance, daidzein and genistein induce *nod* genes in *Bradyrhizobium japonicum*, but inhibit their expression in *Sinorhizobium meliloti*. Similarly, naringenin stimulates Nod formation in *Rhizobium leguminosarum*, but quercetin represses its production. The mechanism of action is through binding of flavonoids to bacterial NodD proteins, members of the transcription factor LysR family [33]. These proteins respond to different flavonoid groups, and a specificity in the interaction between legumes and the nitrogen fixing bacteria can be observed [34]. Activated NodD protein binds to strongly conservative nod-box motifs in the promoter regions of *nod* genes and induces their transcription [35]. As a result, Nod factors (lipopolysaccharides) are synthesized and released by the bacteria, activating various processes in the host plant and leading to preparation of the plant for symbiosis with the bacteria. The factor is recognized by Nod factor receptors (NR) localized in the plant cell membrane. The receptors are composed of an extracellular domain with two or three lysine motifs (LysM) and an intracellular kinase domain (LysM-RLK) [36,37,38,39,40]. Perception of Nod factors requires dimers of LysM [41]. After binding of Nod factors, the receptor proteins start a signaling kinase cascade, in which DMI1, DMI2, NFP and NSP proteins participate [42]. One of the earliest plant reactions in response to Nod factors is the change in intracellular calcium level and in the root hair cell cytoskeleton [33,43], leading to root cell divisions, root nodule formation and infection threads [33]. In addition, rhizobial Nod factors induce the expression of flavonoid synthesis genes. It was proposed that the flavonoids interfere subsequently with auxin transport, thereby promoting cell divisions [44] (Figure 2).

The flavonoids are also very important in the formation of mycorrhiza, which is a symbiotic relationship between a plant and soil-borne fungi that colonize the cortical tissues of roots. Though the role of flavonoids in this symbiosis has not yet been fully recognized, they can have both negative and positive effects on the mycorrhiza [45]. Elevated flavonoid biosynthesis during mycorrhiza development was found in *Trifolium repens* (white clover) [46], melon roots [47] and *Medicago truncatula* [48]. It was demonstrated that mycorrhizal formation changed the flavonoid profile in root extracts by modifying the expression of the genes involved in phenylpropanoid biosynthesis. It was also reported that flavonoids, such as quercetin, quercetin galactoside and kaempferol, have a positive effect on the growth of hyphae and spore germination of mycorrhizal fungi [49,50]. Rutin was found in *Eucalyptus globulus* spp. *bicostata* to promote hyphae of *Pisolithus* sp*.* [51].

## 4. Participation of Flavonoids in Allelopathic Interactions between Plants

Plant-plant interactions can be positive or negative and may depend on the concentrations of the flavonoids [52,53]. The negative relations are mainly based on inhibiting germination and growth of other plants’ seedlings [23]. Quite often, flavonoids are excreted through roots in to the soil where they inhibit seed germination, but can be also found in leaves and even in pollen, which, after falling onto the surrounding soil, inhibits the germination of other plants [54,55]. Some examples include: catechins excreted by *Centaurea maculosa* inhibiting germination and growth of *Centaurea diffusa* and *Arabidopsis thaliana*; and flavones excreted by barley inhibiting weed seed germination [56,57]. Unfortunately, the precise mechanism by which flavonoids participate in allelopathy is still unknown. Potential ways in which they can influence allelopathy may include cell growth inhibition, ATP production disturbances and hindering the proper functioning of auxins [58]. Flavanols were reported to provoke a wave of ROS, which activates the Ca^2+^ signal cascade and root system death [59]. The significance of allelopathy gains more and more attention in agriculture, because these interactions could be employed for reducing weed growth [60,61,62].

## 5. Flavonoids and Plant Pathogen Resistance

Flavonoids are very important in plant resistance against pathogenic bacteria and fungi. Antipathogenic properties of flavonoids can be non-specific and result, in part, from their antioxidative properties. They quench ROS, which are generated both by the pathogens and the plant as a result of the infection [63,64]. Flavonoid compounds are transported to the site of infection and induce the hypersensitivity reaction, which is the earliest defense mechanism employed by the infected plants, and programmed cell death. It was shown that flavonoids are incorporated into the cell walls of necrotic and adjacent cells [63,64,65]. Moreover, flavonoids can contribute to tightening of the plant structures and tissues by modulating auxin (IAA) activity, which can lead to the differentiation of tissues, promotion of callus and tylose formation and closure of the vascular system to prevent pathogen infection [65]. They may also be directly involved in the inhibition of the pathogen’s enzymes, especially those digesting the plant cell wall, by chelating metals required for their activity [23].

The antifungal activity is often based on the inhibition of spore development and mycelium hyphae elongation [63]. Flavonoid antipathogenic activity can also be more specific. It is suggested that the mechanism of flavonoid antibacterial activity is based on their ability to inactivate microbial adhesion and cell envelope transport proteins [66,67]. Fat-soluble flavonoids may also disrupt microbial membranes, change their fluidity and may disturb the respiratory chain [68,69].

Moreover, the B ring of flavonoids can intercalate or form hydrogen bonds with the stacking of nucleic acid bases and further lead to inhibition of DNA and RNA synthesis in bacteria and influence DNA gyrase activity [70]. This is presumably also the basis of their antiviral properties, as they can inhibit viral polymerases and bind to the nucleic acids or capsid proteins of a virus [71].

It was shown that the anti-pathogenic effect of flavonoids depends on their structure. It was suggested that the strongest antifungal activity is demonstrated by unsubstituted flavones and unsubstituted flavanones. Hydroxyl and methyl groups in these compounds reduce their antifungal properties [72], though in some cases, methylated flavonoids reveal a stronger antifungal effect [73]. Flavonoids inhibit a number of root pathogens, especially fungal ones, and in general, isoflavones, flavanes and flavanones are acknowledged as efficient anti-microbial agents [74]. Studies on barley mutants showed that proanthocyanidins or even small amounts of dihydroquercetin are involved in the protection against *Fusarium* sp. This may result from several mechanisms of action, involving cross-linking of microbial enzymes, inhibition of pathogen cellulases, xylanases and pectinases, chelation of metal ions relevant for enzymatic activities and/or tightening of cell walls, leading to the formation of a physical barrier against pathogen attack [75]. Antimicrobial activities of various classes of flavonoids are summarized in Table 1.

**Table 1:** List of flavonoid compounds of anti-pathogenic activities found in plant organisms.

Plant Organism	Antimicrobial Compound	Pathogen	Reference
*Dianthus caryophyllus*	flavonol triglycoside of kaempferide	*Fusarium oxysporum*	[76]
*Dianthus caryophyllus*	kaempferol-*O*-rutinoside, kaempferol-3-*O*-b-d-glucopyranosyl	*Fusarium oxysporum*	[77]
*Linum usitatissimum*	isoorientin, isovitexin, vitexin	*Fusarium. oxysporum*, *Fusarium culmorum*	[78]
*Triticum L. cv. Roblin*	flavonoids	*Fusarium graminearum*	[79]
*Lotus garcinii*	catechin, epicatechin, rutin	*Fusarium graminearum*	[80]
Wheat NILs	5,6-dimethoxyflavone, 2-hydroxyisoflavanone, naringenin, naringenin 7-*O*-b-d-glucoside, 5-hydroxy-7,8-dimethoxyflavanone 5-rhamnoside, kaempferol 3-rhamnoside-7-xylosyl-(1-2)-rhamnoside	*Fusarium graminearum*	[81]
*Hordeum vulgare*	naringenin, kaempferol	*Gibberella zeae*	[82]
*Mariscus psilostachys*	chalcones	*Cladosporium cucumerinum*	[83]
*Mariscus psilostachys*	flavans	*Cladosporium cucumerinum*	[84]
*Arabidopsis thaliana*	quercetin	*Neurospora crassa*	[85]
*Eucalyptus globules*	flavonols	*Cytonaema* sp.	[86]
*Cicer bijugum*	isoflavonoids	*Botrytis cinerea*	[87]
*Medicago truncatula*	isoflavone	*Erysiphe pisi*	[88]
*Vitis vinifera*	quercetin-3-*O*-glucoside	*Plasmopara viticola*	[89]
*Phaseolus vulgaris*	isoflavonoid phytoalexin: genistein, daidzein, 2-hydroxygenistein, dalbergioidin, phaseollin, phaseollidin, phaseollin isoflavan, kievitone, coumestrol	*Colletotrichum lindemuthianum*	[90]
*Austrian pine*	flavonoids	*Diplodia pinea*	[91]
*Glycine max*	isoflavone	*Phytophthora sojae*	[92]
*Solanum tuberosum*	glucosylated forms of flavonoids	*Erwinia carotovora*	[93]
*Oryza sativa*	naringenin, kaempferol, quercetin, hydroxyquercetin	*Xanthomonas oryzae* pv*. oryzae*, *Pyricularia oryzae*	[94]
*Brassica rapa*	kaempferol glucoside	*Xanthomonas campestris* pv.* campestris*	[95]
*Nicotiana tabacum*, *Arabidopsis thaliana*	jaceosidin fisetin hydrate	*Pectobacterium carotovorum*, *Pseudomonas syringae*	[96]
*Lycopersicon esculentum*	flavonoids	*Pseudomonas syringae*	[97]
*Citrus sinensis*	flavonoid glycosides, polymethoxylated flavones	*Candidatus* *Liberibacter*	[98]

## 6. Flavonoids and Environmental Conditions

Due to the inability to move, plants have developed mechanisms to cope with unfavorable environmental conditions. One of those mechanisms is synthesis of secondary metabolites, including flavonoids. The levels of these compounds increase in response to various factors, such as strong light, ultraviolet (UV) radiation, low/high temperature, ozone, heavy metals, drought, *etc*. These conditions are stressful for the plant and are a source of free radicals. One of the functions of flavonoids during oxidative stress triggered by environmental factors is the diminution of the effect caused by the presence of ROS [23,99].

Flavonoids protect plants against UV damage, which, to some extent, results from the fact that they can act as a screen absorbing UV radiation and, as they are accumulated mainly in the epidermis and hypodermis of leaves and stems, apical meristem and pollen, reducing the penetration of UV light to the vulnerable tissues or organs. Besides UV absorption, flavonoid compounds may also transfer or accept light energy to or from other molecules via sensitization [100]. The role of flavonoids in response to UV radiation is mostly due to the scavenging of ROS generated during irradiation. Flavonoids reacting to light are the dihydroxy B-ring-substituted forms, such as quercetin 3-*O* and luteolin 7-*O*-glycosides, and not the monohydroxy B-ring-substituted counterparts, such as apigenin 7-*O*-glycosides and kaempferol 3-*O*-glycosides. Dihydroxy B-ring-substituted flavonoids possess higher antioxidative properties, but lower UV absorption capacity than their monohydroxy B-ring-substituted counterparts. The ratios of quercetin to the kaempferol derivatives or luteolin to the apigenin derivatives drastically increase upon exposure to UV-B, UV-A + UV-B or photosynthetic active radiation (PAR) [101,102]. Synthesis of flavonoids and other phenolic compounds in response to increased UV radiation increases strongly [103,104]. It is probably the mechanism of the primary response of the plant to stressful conditions, which is subsequently followed by other mechanisms, such as pigment accumulations or lignification processes [105]. The regulation of flavonoid biosynthesis proceeds at the transcription level and requires the co-operation of UV-b photoreceptor [105]. Activation of the UV-b photoreceptor leads to triggering of transcription factors, which bind to specific sites on the promoters of flavonoid synthesis genes [106]. Stress factors can induce increased generation of toxic reactive oxygen species (ROS). They are also produced during normal physiological plant activity, but they are strictly controlled by the plant antioxidative system. ROS react non-specifically with lipids, proteins and nucleic acids; thus their increased generation can lead to impairment of cell structures, including cell membranes and the photosynthetic apparatus [103,104,107]. Flavonoids can undergo single electron oxidation and, thus, are capable of reducing free radicals. They act as antioxidants by direct quenching free radicals through transferring a proton from the A and/or B ring and generating less active flavonoid radicals. The capability to quench free radicals is connected with the structure of flavonoids, and the presence, position and modifications of hydroxyl groups in the A and B rings are essential (e.g., methylation or glycosylation reduces the antioxidative capacity of flavonoids, while double bond between C-2 and C-3 linked to 4-keto and 3-hydroxyl groups facilitate electron transfer) [108,109,110]. The spatial arrangement of the B ring can also contribute to efficient electron transfer and influence the antioxidative properties of flavonoids [111]. The ability to quench free hydroxyl radicals increases with the number of hydroxyl groups in the B ring, e.g., myricetin is a stronger antioxidant than kaempferol. One of the best ROS quenchers is quercetin, which contains a catechol group in the B ring, double bond between C2 and C3 and a hydroxyl group at C-3 [108]. Metals are involved in ROS formation in the Fenton reaction [112]. Flavonoids are able to chelate Fe^2+^, Fe^3+^, Cu^2+^, Zn^2+^, Al^3+^ and Mg^2+^ cations, but are unable to bind Na^+^, K^+^ and Ca^2+^ [113]. Metals bind to the flavonoid catechol group localized within the B ring, to the 3-hydroxyl and 4-oxo group of the heterocyclic ring and to the 4-oxo and 5-hydroxyl group between the heterocyclic and A rings [114]. In this way, they can stop lipid peroxidation dependent on Fe^2+^ and Fe^3+^ [108]. Lipid peroxidation is a particularly dangerous process, because it does not conclude with the oxidation of the first constituent, but leads to a chain reaction. It was shown that flavonoids are located in the membrane layer between the lipid bilayer and aqueous phase and can influence both enzymatic and non-enzymatic peroxidation of lipids [115]. It was reported that flavonoids also directly interact with biological membranes, reducing their fluidity, making them more resistant to many oxidative factors and hampering diffusion of free radicals [8,11].

Flavonoids also prevent oxygen radical formation through inhibiting the activity of the enzymes involved in their generation, such as cyclooxygenase, lipoxygenase, microsomal monooxygenase, glutathione S-transferase and xanthine oxidase [8,116]. Hydroxyl groups at C-5 and C-7 and double bonds between C-2 and C-3 are responsible for the ability of flavonoids to inhibit xanthine oxidase activity. The presence of a hydroxyl group at C-3 reduces this ability. Flavanones, dihydroflavonols and flavanols, which contain this group at C-3, are unable to inhibit the activity of xanthine oxidase [111,117,118]. Flavonoids also reduce the activity of membrane NADPH oxidase, which participates in generating the superoxide anion radical [119].

Regulation of gene expression provides a complex control mechanism by which plants respond to abiotic and biotic stresses and modulate developmental processes. One of the elements facilitating gene expression is the activity of transcription factors (TF). Central to the direct regulation of flavonoid biosynthesis genes are core “MBW” regulation complexes, comprising specific members of the R2R3MYB and basic helix-loop-helix (bHLH) TF families, which are responsible for binding specific regulatory sequences and WD-repeat factor (WDR; tryptophan-aspartic acid (W-D) dipeptide repeat). Variant MBW complexes can form from different MYB and bHLH components, and these can have different target genes and vary in their activation or repression actions. The WDR protein is common to all of the variant MBW complexes. The bHLH component may also be common to MBW complexes targeting different biosynthetic pathways. However, distinct R2R3MYBs are involved in regulating the different branches of flavonoid production [120,121,122,123]. Members of the MYB transcription factor superfamily are characterized by the presence of an amino acid motif structurally and functionally related to the DNA-binding domain of the product of the retroviral oncogene v-MYB and its animal cellular counterpart, c-MYB [120,123]. MYB regulators are divided into four types: MYB1R, R2R3-MYB, R1R2R3 MYB (MYB 3R) and 4RYB [124,125]. MYB proteins have been identified in a large number of eukaryotic organisms. In many plant species, MYB proteins were reported to be capable of regulating the biosynthesis of flavonoids and to be induced by stress, exemplified by *Arabidopsis* MYB75 (PAP1), AtMYB90 (PAP2) MYB12 and MYBL2 [120,126,127], petunia AN2 and PH4 [128,129], grape MYBA1 and MYBA2 [130,131,132,133], sweet potato [134], apple MYB10/MYB1/MYBA [135,136], legume LAP1 [137] and persimmon MYB4 [138] and *Epimedium sagittatum* MYBA1 [139].

**Figure 3:**
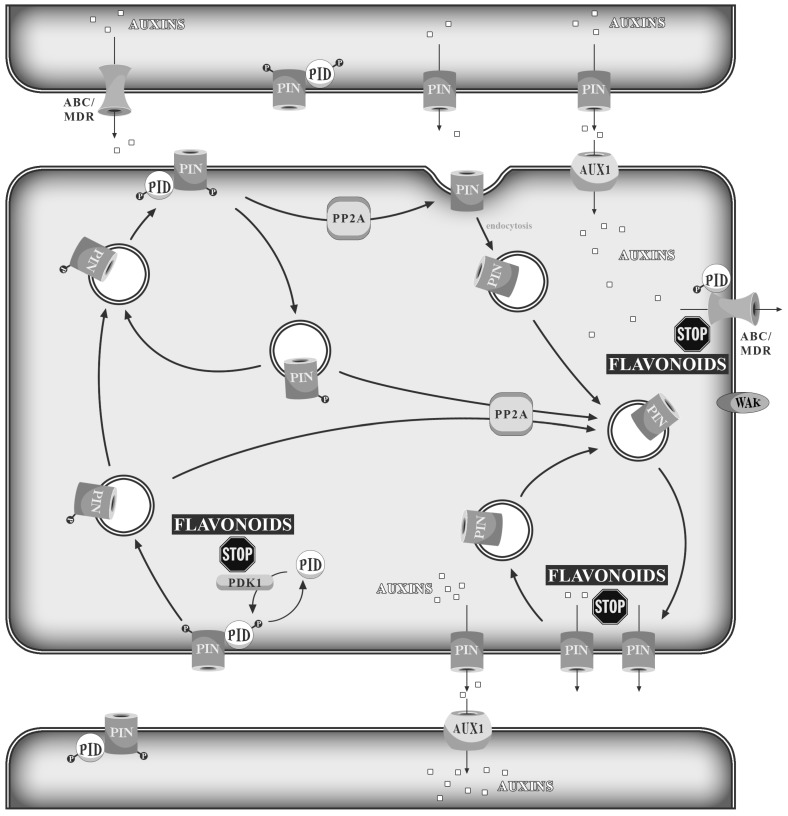
Flavonoids’ interference with PIN and MDR/ABC proteins, as well as with PID, WAK and PDK1 kinases leads to impaired auxin transport.

Flavonoids can modulate the plant response to stresses by controlling the process of auxin transport. Auxins are one of the most important phytohormones and influence such processes as root development, mitotic transition and gene transcription. The response to hormones is connected with specific receptors, but integration and coordination between the hormone and the signaling pathway require an integrator. Flavonoids can constitute such an integrator for auxins [140,141]. Jacobs and Rubery (1988) found that flavonoids compete for auxin transporters with 1-naphthylphthalamic acid (NPA) *in vitro* [142], which suggests that flavonoids bind to NPA-interacting proteins. Studies in *Arabidopsis* suggest that these could be either plasma membrane aminopeptidase or multi-drug resistant (MDR) ABC type transporters. *Arabidopsis* mutants with reduced flavonoid contents show elevated auxin transport levels and phenotype changes, architectonic abnormalities and gravitropic disorders [143,144,145,146]. Two NPA-binding protein complexes were found, one with a low- and one with a high-affinity NPA binding site. The first one contained a flavonol-sensitive aminopeptidase, AtAPM1, localized at the plasma membrane [147], while the second included different proteins homologous to human MDR/ABC transporters [147,148]. Additionally, the asymmetric distribution of the PIN-FORMED 1–4 (PIN1–4) auxin efflux proteins surrounding the plasma membrane regions probably contribute to auxin polar transport [149,150,151,152]. Flavonoids are efficient inhibitors of glycoproteins of PIN and MDR, which are involved in intercellular auxin transport. The ability of flavonoids to inhibit the PIN and MDR proteins correlates with the presence of a catechol group in the flavonoid molecule scaffold. Quercetin is a stronger inhibitor of auxin polar transport than kaempferol [153,154]. A small amount of auxins is transported from cell to cell through diffusion, but the majority of auxins exist in anionic form and require specific carriers for directed transport, which is crucial for the local gradient of this phytohormone. One of the mechanisms controlling this transport involves reversible protein phosphorylation by the protein kinase, PINOID (PIN) [155]. This kinase has been found in the primordia of cotyledons, leaves and floral organs and in vascular tissue in developing organs or proximal to meristems [156]. PID belongs to AGCVIII-specific plant kinases and regulates asymmetric subcellular localization of the PIN-FORMED (PIN) auxin efflux facilitator protein responsible for the auxin gradient [157]. PID plays the role of a switch influencing the PIN protein distribution in the cell membrane [158,159]. The activity of PID is regulated by PDK1 protein kinase [160]. PID partially co-localizes with PIN. PIN and MDR glycoprotein control, separately or jointly, auxin cell-to-cell transport [161,162]. Flavonoids can regulate auxin efflux by interfering with the membrane PIN/MDR glycoproteins, PDK1 and a wide range of cell wall-associated kinases (WAKs). The WAKs allow cells to recognize the extracellular environment and react by altering the cell shape (Figure 3) [163]. Control of auxin transport by flavonoids can be important in the stress-induced morphogenetic response of plants (SIMR). Species rich in dihydroxy-flavonoids present phenotypes of strikingly different morphological features in comparison to those rich in monohydroxy-flavonoids [164,165]. Branchy phenotypes are usual in insolated environments and have a few small, thick leaves exposed directly to the sunlight, thus protecting the leaves below. In contrast, shady plants rich in kaempferol and/or apigenin derivatives have long internodes and a large, thin leaf blade [165]. SIMR can contribute to the decreased damage of tissues or organs caused by solar radiation.

Flavonoids can also regulate the activity of IAA-oxidase, with different effects depending on their chemical structure [166,167].

Besides the protective functions, flavonoids can increase the availability of nutritional elements. During the period of low nutrition availability, flavonoids are released to the soil with the help of ABC transporters, where they can bind metals necessary for plant growth [168,169]. For example, isoflavonoids excreted by roots of *Medicago sativa* L. increase the availability of iron cations and phosphate anions [170]. Genistein, quercetin and kaempferol change the availability of iron by reducing Fe^3+^ to Fe^2+^ and chelating the unavailable iron from iron oxides [171].

## 7. Prospects for the Practical Application of Flavonoids

In order to improve the efficacy of agricultural production, plant protection chemicals are widely employed. However, such chemicals are not neutral for the environment, especially for living organisms, including humans. Growing emphasis is being placed on the use of safer biopesticides. Flavonoids appear to be ideal candidates as constituents of such biopesticides. Maysin is a naturally occurring C-glycosyl flavone found in maize (*Zea mays* L.) silk tissue that confers resistance to corn earworm (*Helicoverpa zea*, Boddie). Transgenic maize with elevated quantities of this compound showed higher resistance towards larvae of the earworm. Lower body weight of the larvae and their higher mortality were observed [172]. Cespedes *et al.* found that extracts from *Calceolaria* that contained secondary metabolites, including flavonoids, revealed insecticidal properties against *Spodoptera frugiperda* and *Drosophila melanogaster*. The authors also observed antibacterial and antifungal effects of these extracts. *Fusarium* is known to cause the greatest losses in flax cultivation. Genetic engineering, combined with traditional cross-breeding, has led to the production of new plants resistant to this pathogen [78]. Many other examples of more resistant plants overexpressing flavonoids are summarized in Table 1.

Weeds are a major problem in agriculture and are difficult to eradicate. Biopesticides based on flavonoids displaying allelopathic properties against weeds can potentially be an efficient natural defense against them. Biological trials performed by Kong *et al.* showed inhibiting activity against weeds of the species *Echinochloa crus-galli*, *Cyperus difformis* and *Cyperus iria* of 5,7,4'-trihydroxy-3',5'-dimethoxyflavone, which additionally displayed an antifungal effect [57]. Phytotoxic effects were also observed for flavonoids from *Stellera chamaejasme* L. [173]. In agriculture, flavonoids have also found application in floriculture. A number of genetic modifications of the flavonoid pathway have been described, which led to the production of new colors of flowers in such species as petunia, gerbera, rose, carnation, lisianthus and *Torenia* [174,175,176].

Many flavonoids are characterized by antibacterial, antifungal and antiviral activities, not only against plant pathogens, but also against the pathogens dangerous for humans. For instance, apigenin and amentoflavone display strong effects against the pathogenic fungi *C. albicans*,* S. cerevisiae* and *T. beigelii*. Kaempferol shows activity against Gram-positive and Gram-negative bacteria, as well as against the fungus *Candida glabrata* [177]. Studies also show that flavonoids can be active against antibiotic resistant strains [177]. Flavonoids, as compounds originating from plants, are part of the human diet and have many positive impacts on the human organism. They act as natural antioxidants and have an effect on many diseases. They have anti-tumor [122,178,179], anti-inflammatory [180,181], anti-allergic [182], anti-thrombotic [183], anti-diabetic [184] and anti-atherosclerotic activities [185]. A wide range of activities of flavonoid compounds is employed in cosmetology. Flavonoids improve skin hydration, restore its antibacterial barrier, smoothen its surface and induce skin cells to grow. They have protective, astringent and antiedema properties. They are also used in the treatment of acne, blackheads and dandruff, prevent baldness and wrinkles and slow down the ageing processes [186].

Transgenic plants with overproduction of flavonoids can be used in the production of plant-based medical products. Fiber, seedcake extract and oil from transgenic flax with overexpression of three genes of flavonoid synthesis became a starting point for the production of dressings for chronic wounds and ulcerations. Such wound dressings caused diminution of wound size, accelerated the wound healing process and reduced the pain connected with long lasting wounds [187].

Due to their wide range of activities, flavonoids can be used in agriculture, but also in medicine, pharmacy and cosmetology. They are an increasingly attractive group of compounds, being competitive with those used so far, mainly due to the fact that they are natural and display no adverse effects.

## 8. Summary

In conclusion, flavonoids are molecules displaying various biological activities with relevance to plant physiology and development. Flavonoids not only participate in protection against harmful abiotic factors, but also allow for interactions with other plants and microorganisms. Previously, the role of flavonoids was mostly explained in terms of their antioxidative properties; however, due to recent research, they are also emerging as regulatory and signaling molecules. In particular, their influence on plant development via interaction with an auxin transport network points toward their regulatory function in view of very low concentrations needed for the activity, indeed much lower than is necessary for effective radical quenching. Their presence in the nucleus points towards their role as transcriptional regulators [188,189]. This function is already performed in regulation of microbial genes during mycorrhizal interactions. The effect of flavonoids on cellular signaling is well described in animal models where dietary flavonoids interact with many proteins of signaling cascades by directly binding to the ATP catalytic sites of protein kinases [139]. The possible interactions with MAP kinases in plants are still unexplored; however, it is likely that flavonoids may be responsible for mediating ROS-induced signaling cascades vital to cell growth and differentiation.

It is evident that flavonoids allow plants to be an integrative part of their environment by responding to biotic and abiotic stimuli. Although so much is known about the roles of these secondary metabolites, the knowledge is still incomplete and requires extensive research. Expanding knowledge on this topic and ways to manipulate flavonoid contents should allow for more practical use of these chemicals in agriculture, industry and medicine.

## References

[1] Buer C.S., Imin N., Djordjevic M.A. (2010). Flavonoids: New roles for old molecules. J. Integr. Plant Biol..

[2] Bohm B. (1998). Introduction of Flavonoids.

[3] Forkmann G., Heller W. (1999). Biosynthesis of flavonoids. Comprehensive Natural Products Chemistry.

[4] Rice-Evans C.A., Miller N.J., Paganga G. (1996). Structure-antioxidant activity relationships of flavonoids and phenolic acids. Free Radic. Biol. Med..

[5] Seyoum A., Asres K., El-Fiky F.K. (2006). Structure-radical scavenging activity relationships of flavonoids. Phytochemistry.

[6] Amić D., Davidović-Amić D., Bešlo D., Trinajstić N. (2003). Structure-radical scavenging activity relationships of flavonoids. Croat. Chem. Acta.

[7] Cotelle N., Bernier J.L., Catteau J.P., Pommery J., Wallet J.C., Gaydou E.M. (1996). Antioxidant properties of hydroxy-flavones. Free Radic. Biol. Med..

[8] Harborne J.B., Williams C.A. (2000). Advances in flavonoid research since 1992. Phytochemistry.

[9] Higdon J.V., Frei B. (2003). Tea catechins and polyphenols: Health effects, metabolism, and antioxidant functions. Crit. Rev. Food Sci. Nutr..

[10] Jovanovic S.V., Steeden S., Tosic M., Marjanovic B., Simic G.M. (1994). Flavonoids as antioxidants. J. Am. Chem. Soc..

[11] Arora A., Byrem T.M., Nair M.G., Strasburg G.M. (2000). Modulation of liposomal membrane fluidity by flavonoids and isoflavonoids. Arch. Biochem. Biophys..

[12] Buettner G.R. (1993). The pecking order of free radicals and antioxidants: Lipid peroxidation, alpha-tocopherol, and ascorbate. Arch. Biochem. Biophys..

[13] Koes R.E., Quattrocchio F. (1994). The flavonoid biosynthetic pathway in plants: Function and evolution. BioEssays.

[14] Ohsugi T., Nishida R., Fukami H. (1985). Oviposition stimulant of Papilio xuthus, a Citrus feeding swallowtail butterfly. Agric. Biol. Chem..

[15] Feeny P., Sachdev K., Rosenberry L., Carter M. (1988). Luteolin 7-*O*-(6''-O-malonyl)-β-d-glucoside and trans-chlorogenic acid: Oviposition stimulants for the black swallowtail butterfly. Phytochemistry.

[16] Nishida R. (1994). Oviposition stimulant of a Zeryntiine swallowtail butterfly, Luehdorfia japonica. Phytochemistry.

[17] Tabashnik B.E. (1987). Plant secondary compounds as oviposition deterrents for cabbage butterfly, Pieris rapae (Lepidoptera: Pieridae). J. Chem. Ecol..

[18] War A.R., Paulraj M.G., Ahmad T., Buhroo A.A., Hussain B., Ignacimuthu S., Sharma H.C. (2012). Mechanisms of plant defense against insect herbivores. Plant Signal. Behav..

[19] Nuessly G.S., Scully B.T., Hentz M.G., Beiriger R., Snook M.E., Widstrom N.W. (2007). Resistance to Spodoptera frugiperda (Lepidoptera: Noctuidae) and Euxesta stigmatias (Diptera: Ulidiidae) in sweet corn derived from exogenous and endogenous genetic systems. J. Econ. Entomol..

[20] Simmonds M.S. (2003). Flavonoid-insect interactions: Recent advances in our knowledge. Phytochemistry.

[21] Simmonds M.S., Stevenson P.C. (2001). Effects of isoflavonoids from Cicer on larvae of Heliocoverpa armigera. J. Chem. Ecol..

[22] Sosa T., Chaves N., Alias J.C., Escudero J.C., Henao F., Gutierrez-Merino C. (2004). Inhibition of mouth skeletal muscle relaxation by flavonoids of *Cistus ladanifer* L.: A plant defense mechanism against herbivores. J. Chem. Ecol..

[23] Treutter D. (2005). Significance of flavonoids in plant resistance and enhancement of their biosynthesis. Plant Biol..

[24] Gould K.S., Lister C. (2006). Flavonoid functions in plants. Flavonoids: Chemistry, Biochemistry, and Applications.

[25] Mallikarjuna N., Kranthi K.R., Jadhav D.R., Kranthi S., Chandra S. (2004). Influence of foliar chemical compounds on the development of Spodoptera litura in interspecific derivatives of groundnut. J. Appl. Entomol..

[26] Wuyts N., Swennen R., de Waele D. (2006). Effects of plant phenylpropanoid pathway products and selected terpenoids and alkaloids on the behaviour of the plant-parasitic nematodes Radopholus similis, Pratylenchus penetrans and Meloidogyne incognita. Nematology.

[27] Webster G., Jain V., Davey M.R., Gough C., Vasse J., Dénarié J., Coking E.C. (1998). The flavonoid naringenin stimulates the intercellular colonization of wheat roots by Azorhizobium caulinodans. Plant Cell Environ..

[28] Hartwig U.A., Maxwell C.A., Joseph C.M., Phillips D.A. (1990). Chrysoeriol and luteolin released from alfalfa seeds induce nod genes in Rhizobium meliloti. Plant Physiol..

[29] Abdel-Lateif K., Bogusz D., Hocher V. (2012). The role of flavonoids in the establishment of plant roots endosymbioses with arbuscular mycorrhiza fungi, rhizobia and Frankia bacteria. Plant Signal. Behav..

[30] Denarie J., Debelle F., Prome J.C. (1996). Rhizobium lipo-chitooligosaccharide nodulation factors: Signaling molecules mediating recognition and morphogenesis. Annu. Rev. Biochem..

[31] Cooper J.E. (2004). Multiple responses of rhizobia to flavonoids during legume root infection. Adv. Bot. Res..

[32] Reddy P.M., Rendón-Anaya M., de los Dolores Soto del Rio M., Khandual S. (2007). Flavonoids as signalling molecules and regulators of root nodule development. Dyn. Soil Dyn. Plant.

[33] Jones K.M., Kobayashi H., Davies B.W., Taga M.E., Walker G.C. (2007). How rhizobial symbionts invade plants: The Sinorhizobium-Medicago model. Nat. Rev. Microbiol..

[34] Perret X., Staehelin C., Broughton W.J. (2000). Molecular basis of symbiotic promiscuity. Microbiol. Mol. Biol. Rev..

[35] Rostas K., Kondorosi E., Horvath B., Simoncsits A., Kondorosi A. (1986). Conservation of extended promoter regions of nodulation genes in Rhizobium. Proc. Natl. Acad. Sci. USA.

[36] Madsen E.B., Madsen L.H., Radutoiu S., Olbryt M., Rakwalska M., Szczyglowski K., Sato S., Kaneko T., Tabata S., Sandal N. (2003). A receptor kinase gene of the LysM type is involved in legume perception of rhizobial signals. Nature.

[37] Radutoiu S., Madsen L.H., Madsen E.B., Felle H.H., Umehara Y., Gronlund M., Sato S., Nakamura Y., Tabata S., Sandal N. (2003). Plant recognition of symbiotic bacteria requires two LysM receptor-like kinases. Nature.

[38] Arrighi J.F., Barre A., Ben Amor B., Bersoult A., Soriano L.C., Mirabella R., de Carvalho-Niebel F., Journet E.P., Gherardi M., Huguet T. (2006). The Medicago truncatula lysin [corrected] motif-receptor-like kinase gene family includes NFP and new nodule-expressed genes. Plant Physiol..

[39] Lohmann G.V., Shimoda Y., Nielsen M.W., Jorgensen F.G., Grossmann C., Sandal N., Sorensen K., Thirup S., Madsen L.H., Tabata S. (2010). Evolution and regulation of the Lotus japonicus LysM receptor gene family. Mol. Plant-Microbe Interact..

[40] Limpens E., Franken C., Smit P., Willemse J., Bisseling T., Geurts R. (2003). LysM domain receptor kinases regulating rhizobial Nod factor-induced infection. Science.

[41] Gough C., Cullimore J. (2011). Lipo-chitooligosaccharide signaling in endosymbiotic plant-microbe interactions. Mol. Plant-Microbe Interact..

[42] Smit P., Limpens E., Geurts R., Fedorova E., Dolgikh E., Gough C., Bisseling T. (2007). Medicago LYK3, an entry receptor in rhizobial nodulation factor signaling. Plant Physiol..

[43] Gage D.J. (2004). Infection and invasion of roots by symbiotic, nitrogen-fixing rhizobia during nodulation of temperate legumes. Microbiol. Mol. Biol. Rev..

[44] De Billy F., Grosjean C., May S., Bennett M., Cullimore J.V. (2001). Expression studies on AUX1-like genes in Medicago truncatula suggest that auxin is required at two steps in early nodule development. Mol. Plant-Microbe Interact..

[45] Nair M.G., Safir G.R., Siqueira J.O. (1991). Isolation and identification of vesicular-arbuscular mycorrhiza-stimulatory compounds from clover (Trifolium repens) Roots. Appl. Environ. Microbiol..

[46] Ponce M.A., Scervino J.M., Erra-Balsells R., Ocampo J.A., Godeas A.M. (2004). Flavonoids from shoots and roots of Trifolium repens (white clover) grown in presence or absence of the arbuscular mycorrhizal fungus Glomus intraradices. Phytochemistry.

[47] Akiyama K., Matsuoka H., Hayashi H. (2002). Isolation and identification of a phosphate deficiency-induced C-glycosylflavonoid that stimulates arbuscular mycorrhiza formation in melon roots. Mol. Plant-Microbe Interact..

[48] Schliemann W., Ammer C., Strack D. (2008). Metabolite profiling of mycorrhizal roots of Medicago truncatula. Phytochemistry.

[49] Tsai S.M., Phillips D.A. (1991). Flavonoids released naturally from alfalfa promote development of symbiotic glomus spores *in vitro*. Appl. Environ. Microbiol..

[50] Poulin M.J., Simard J., Catford J.G., Labrie F., Piche Y. (1997). Response of symbiotic endomycorrhizal fungi to estrogen and antiestrogens. Mol. Plant-Microbe Interact..

[51] Lagrange H., Jay-Allemand C., Lapeyrie F. (2001). Rutin, the phenolglycoside from eucalyptus root exudates, stimulates Pisolithus hyphal growth at picomolar concentrations. New Phytol..

[52] Chou C.H. (1999). Roles of allelopathy in plant biodiversity and sustainable agriculture. Crit. Rev. Plant Sci..

[53] Inderjit S., Gross E., Martens S., Forkmann G., Treutter D. (2000). Plant phenolics: Potential role in aquatic and terrestrial ecosystems. Proceedings of the Polyphenols 2000: XXth International Conference on Polyphenols.

[54] Star A.E. (1980). Frond exudate flavonoids as allelopathic agents in Pityrogramma. Bull. Torrey Bot. Club.

[55] Cooper-Driver G. (1980). The role of flavonoids and related compounds in fern systematics. Bull. Torrey. Bot. Club.

[56] Kong C.H., Zhao H., Xu X.H., Wang P., Gu Y. (2007). Activity and allelopathy of soil of flavone *O*-glycosides from rice. J. Agric. Food Chem..

[57] Kong C., Xu X., Zhou B., Hu F., Zhang C., Zhang M. (2004). Two compounds from allelopathic rice accession and their inhibitory activity on weeds and fungal pathogens. Phytochemistry.

[58] Berhow M.A., Vaughn S.F., Dakshini K.M.M., Foy C.L. (1999). Higher plant flavonoids: Biosynthesis and chemical ecology. Principles and Practices in Plant Ecology. Allelochemical Interaction.

[59] Bais H.P., Vepachedu R., Gilroy S., Callaway R.M., Vivanco J.M. (2003). Allelopathy and exotic plant invasion: From molecules and genes to species interactions. Science.

[60] Weston L.A., Alsaadawi I.S., Baerson S.R. (2013). Sorghum allelopathy—From ecosystem to molecule. J. Chem. Ecol..

[61] Kato-Noguchi H., Peters R.J. (2013). The role of momilactones in rice allelopathy. J. Chem. Ecol..

[62] Schulz M., Marocco A., Tabaglio V., Macias F.A., Molinillo J.M. (2013). Benzoxazinoids in rye allelopathy—from discovery to application in sustainable weed control and organic farming. J. Chem. Ecol..

[63] Blount J.W., Dixon R.A., Paiva N.L. (1992). Stress responses in alfalfa (*Medicago sativa* L.) XVI. Antifungal activity of medicarpin and its biosynthetic precursors; implications for the genetic manipulation of stress metabolites. Physiol. Mol. Plant Pathol..

[64] Dai G.H., Nicole M., Andary C., Martinez C., Bresson E., Boher B., Daniel J.F., Geiger J.P. (1996). Flavonoids accumulate in cell walls, middle lamellae and callose-rich papillae during an incompatible interaction between Xanthomonas campestris pv. malvacearum and cotton. Physiol. Mol. Plant Pathol..

[65] Beckman C.H. (2000). Phenolic-storing cells: Keys to programmed cell death and periderm formation in wilt disease resistance and in general defence responses in plants?. Physiol. Mol. Plant Pathol..

[66] Plaper A., Golob M., Hafner I., Oblak M., Solmajer T., Jerala R. (2003). Characterization of quercetin binding site on DNA gyrase. Biochem. Biophys. Res. Commun..

[67] Naoumkina M.A., Zhao Q., Gallego-Giraldo L., Dai X., Zhao P.X., Dixon R.A. (2010). Genome-wide analysis of phenylpropanoid defence pathways. Mol. Plant Pathol..

[68] Mishra A.K., Mishra A., Kehri H.K., Sharma B., Pandey A.K. (2009). Inhibitory activity of Indian spice plant Cinnamomum zeylanicum extracts against Alternaria solani and Curvularia lunata, the pathogenic dematiaceous moulds. Ann. Clin. Microbiol. Antimicrob..

[69] Haraguchi H., Tanimoto K., Tamura Y., Mizutani K., Kinoshita T. (1998). Mode of antibacterial action of retrochalcones from Glycyrrhiza inflata. Phytochemistry.

[70] Wu T., Zang X., He M., Pan S., Xu X. (2013). Structure-activity relationship of flavonoids on their anti-Escherichia coli activity and inhibition of DNA gyrase. J. Agric. Food Chem..

[71] Selway J.W. (1986). Antiviral activity of flavones and flavans. Prog. Clin. Biol. Res..

[72] Weidenborner M., Jha H.C. (1994). Antifungal activity of flavonoids in relation to degree of hydroxylation, methoxylation and glycosidation. Acta Hortic..

[73] Christensen A.B., Gregersen P.L., Schroder J., Collinge D.B. (1998). A chalcone synthase with an unusual substrate preference is expressed in barley leaves in response to UV light and pathogen attack. Plant Mol. Biol..

[74] Makoi J.H.J.R., Ndakidemi P.A. (2007). Biological, ecological and agronomic significance of plant phenolic compounds in rhizosphere of the symbiotic legumes. Afr. J. Biotechnol..

[75] Skadhauge B., Thomsen K., von Wettstein D. (1997). The role of barley testa layer and its flavonoid content in resistance to Fusarium infections. Hereditas.

[76] Curir P., Dolci M., Galeotti F. (2005). A phytoalexin-like flavonol involved in the carnation (Dianthus caryophyllus)—Fusarium oxysporum dianthi pathosystem. J. Phytopathol..

[77] Galeotti F., Barile E., Curir P., Dolci M., Lanzotti V. (2008). Flavonoids from carnation (Dianthus caryophyllus) and their antifungal activity. Phytochem. Lett..

[78] Mierziak J., Wojtasik W., Kostyn K., Czuj T., Szopa J., Kulma A. (2014). Crossbreeding of transgenic flax plants overproducing flavonoids and glucosyltransferase results in progeny of improved antifungal and antioxidative properties. Mol. Breed..

[79] Ravensdale M., Rocheleau H., Wang L., Nasmith C., Ouellet T., Subramaniam R. (2014). Components of priming-induced resistance to Fusarium head blight in wheat revealed by two distinct mutants of *Fusarium graminearum*. Mol. Plant Pathol..

[80] Girardi F.A., Tonial F., Chini S.O., Sobottka A.M., Scheffer-Basso S.M., Bertol C.D. (2014). Phytochemical profile and antimicrobial properties of *Lotus* spp. (Fabaceae). An. Acad. Bras. Cienc..

[81] Gunnaiah R., Kushalappa A.C., Duggavathi R., Fox S., Somers D.J. (2012). Integrated metabolo-proteomic approach to decipher the mechanisms by which wheat QTL (Fhb1) contributes to resistance against Fusarium graminearum. PLoS One.

[82] Bollina V., Kumaraswamy G.K., Kushalappa A.C., Choo T.M., Dion Y., Rioux S., Faubert D., Hamzehzarghani H. (2010). Mass spectrometry-based metabolomics application to identify quantitative resistance-related metabolites in barley against Fusarium head blight. Mol. Plant Pathol..

[83] Gafner S., Wolfender J.L., Mavi S., Hostettmann K. (1996). Antifungal and antibacterial chalcones from Myrica serrata. Planta Med..

[84] Gafner S., Wolfender J.L., Mavi S., Hostettmann K. (1996). Five flavans from Mariscus psilostachys. Phytochemistry.

[85] Parvez M.M., Tomita-Yokotani K., Fujii Y., Konishi T., Iwashina T. (2004). Effects of quercetin and its seven derivatives on the growth of Arabidopsis thaliana and Neurospora crassa. Biochem. Syst. Ecol..

[86] Eyles A., Davies N.W., Yuan Z.Q., Mohammed C. (2003). Host response to natural infection by Cytonaema sp. in the aerial bark of Eucalyptus globulus. For. Pathol..

[87] Stevenson P.C., Haware M.P. (1999). Maackiain in Cicer bijugum Rech. f. associated with resistance to Botrytis grey mould. Biochem. Syst. Ecol..

[88] Foster-Hartnett D., Danesh D., Penuela S., Sharopova N., Endre G., Vandenbosch K.A., Young N.D., Samac D.A. (2007). Molecular and cytological responses of Medicago truncatula to Erysiphe pisi. Mol. Plant Pathol..

[89] Ali K., Maltese F., Figueiredo A., Rex M., Fortes A.M., Zyprian E., Pais M.S., Verpoorte R., Choi Y.H. (2012). Alterations in grapevine leaf metabolism upon inoculation with *Plasmopara viticola* in different time-points. Plant Sci..

[90] Durango D., Pulgarin N., Echeverri F., Escobar G., Quinones W. (2013). Effect of salicylic acid and structurally related compounds in the accumulation of phytoalexins in cotyledons of common bean (*Phaseolus vulgaris* L.) cultivars. Molecules.

[91] Sherwood P., Bonello P. (2013). Austrian pine phenolics are likely contributors to systemic induced resistance against Diplodia pinea. Tree Physiol..

[92] Subramanian S., Graham M.Y., Yu O., Graham T.L. (2005). RNA interference of soybean isoflavone synthase genes leads to silencing in tissues distal to the transformation site and to enhanced susceptibility to Phytophthora sojae. Plant Physiol..

[93] Lorenc-Kukula K., Jafra S., Oszmianski J., Szopa J. (2005). Ectopic expression of anthocyanin 5-*O*-glucosyltransferase in potato tuber causes increased resistance to bacteria. J. Agric. Food Chem..

[94] Padmavati M., Sakthivel N., Thara K.V., Reddy A.R. (1997). Differential sensitivity of rice pathogens to growth inhibition by flavonoids. Phytochemistry.

[95] Velasco P., Lema M., Francisco M., Soengas P., Cartea M.E. (2013). *In vivo* and *in vitro* effects of secondary metabolites against *Xanthomonas campestris* pv. *campestris*. Molecules.

[96] Song G.C., Ryu S.Y., Kim Y.S., Lee J.Y., Choi J.S., Ryu C.M. (2013). Elicitation of induced resistance against *Pectobacterium carotovorum* and *Pseudomonas syringae* by specific individual compounds derived from native Korean plant species. Molecules.

[97] Vargas P., Farias G.A., Nogales J., Prada H., Carvajal V., Baron M., Rivilla R., Martin M., Olmedilla A., Gallegos M.T. (2013). Plant flavonoids target Pseudomonas syringae pv. tomato DC3000 flagella and type III secretion system. Environ. Microbiol. Rep..

[98] Hijaz F.M., Manthey J.A., Folimonova S.Y., Davis C.L., Jones S.E., Reyes-De-Corcuera J.I. (2013). An HPLC-MS characterization of the changes in sweet orange leaf metabolite profile following infection by the bacterial pathogen Candidatus Liberibacter asiaticus. PLoS One.

[99] Tattini M., Galardi C., Pinelli P., Massai R., Remorini D., Agati G. (2004). Differential accumulation of flavonoids and hydroxycinnamates in leaves of Ligustrum vulgare under excess light and drought stress. New Phytol..

[100] Sisa M., Bonnet S.L., Ferreira D., van der Westhuizen J.H. (2010). Photochemistry of flavonoids. Molecules.

[101] Agati G., Azzarello E., Pollastri S., Tattini M. (2012). Flavonoids as antioxidants in plants: Location and functional significance. Plant Sci..

[102] Fini A., Brunetti C., di Ferdinando M., Ferrini F., Tattini M. (2011). Stress-induced flavonoid biosynthesis and the antioxidant machinery of plants. Plant Signal. Behav..

[103] Kanazawa K., Hashimoto T., Yoshida S., Sungwon P., Fukuda S. (2012). Short photoirradiation induces flavonoid synthesis and increases its production in postharvest vegetables. J. Agric. Food Chem..

[104] Xie Y., Xu D., Cui W., Shen W. (2012). Mutation of Arabidopsis HY1 causes UV-C hypersensitivity by impairing carotenoid and flavonoid biosynthesis and the down-regulation of antioxidant defence. J. Exp. Bot..

[105] Jansen M.A.K., Gaba V., Greenberg B.M. (1998). Higher plants and UV-B radiation: Damage, repair and acclimation. Trends Plant Sci..

[106] Greenberg B.M., Wilson M.I., Huang X.-D., Duxbury C.L., Gerhardt K.E., Gensemer R.W. (1997). The effects of ultraviolet-B radiation on higher plants. Plants for Environmental Studies.

[107] Ryan K.G., Swinny E.E., Markham K.R., Winefield C. (2002). Flavonoid gene expression and UV photoprotection in transgenic and mutant Petunia leaves. Phytochemistry.

[108] Arora A., Nair M.G., Strasburg G.M. (1998). Structure-activity relationships for antioxidant activities of a series of flavonoids in a liposomal system. Free Radic. Biol. Med..

[109] Cao G., Sofic E., Prior R.L. (1997). Antioxidant and prooxidant behavior of flavonoids: Structure-activity relationships. Free Radic. Biol. Med..

[110] Heim K.E., Tagliaferro A.R., Bobilya D.J. (2002). Flavonoid antioxidants: Chemistry, metabolism and structure-activity relationships. J. Nutr. Biochem..

[111] Cos P., Ying L., Calomme M., Hu J.P., Cimanga K., van Poel B., Pieters L., Vlietinck A.J., Vanden Berghe D. (1998). Structure-activity relationship and classification of flavonoids as inhibitors of xanthine oxidase and superoxide scavengers. J. Nat. Prod..

[112] Halliwell B., Gutteridge J. (1998). Free Radicals in Biology and Medicine.

[113] Packer L. (2001). Flavonoids and Other Polyphenols: Methods in Enzymology.

[114] Pietta P.G. (2000). Flavonoids as antioxidants. J. Nat. Prod..

[115] Terao J., Piskula M., Yao Q. (1994). Protective effect of epicatechin, epicatechin gallate, and quercetin on lipid peroxidation in phospholipid bilayers. Arch. Biochem. Biophys..

[116] Brown J.E., Khodr H., Hider R.C., Rice-Evans C.A. (1998). Structural dependence of flavonoid interactions with Cu^2+^ ions: Implications for their antioxidant properties. Biochem. J..

[117] Nagao A., Seki M., Kobayashi H. (1999). Inhibition of xanthine oxidase by flavonoids. Biosci. Biotechnol. Biochem..

[118] Ursini F., Maiorino M., Morazzoni P., Roveri A., Pifferi G. (1994). A novel antioxidant flavonoid (IdB 1031) affecting molecular mechanisms of cellular activation. Free Radic. Biol. Med..

[119] Hodnick W.F., Duval D.L., Pardini R.S. (1994). Inhibition of mitochondrial respiration and cyanide-stimulated generation of reactive oxygen species by selected flavonoids. Biochem. Pharmacol..

[120] Mehrtens F., Kranz H., Bednarek P., Weisshaar B. (2005). The Arabidopsis transcription factor MYB12 is a flavonol-specific regulator of phenylpropanoid biosynthesis. Plant Physiol..

[121] Lepiniec L., Debeaujon I., Routaboul J.M., Baudry A., Pourcel L., Nesi N., Caboche M. (2006). Genetics and biochemistry of seed flavonoids. Annu. Rev. Plant Biol..

[122] Falcone Ferreyra M.L., Rius S., Emiliani J., Pourcel L., Feller A., Morohashi K., Casati P., Grotewold E. (2010). Cloning and characterization of a UV-B-inducible maize flavonol synthase. Plant J..

[123] Verdier J., Zhao J., Torres-Jerez I., Ge S., Liu C., He X., Mysore K.S., Dixon R.A., Udvardi M.K. (2012). MtPAR MYB transcription factor acts as an on switch for proanthocyanidin biosynthesis in Medicago truncatula. Proc. Natl. Acad. Sci. USA.

[124] Dubos C., Stracke R., Grotewold E., Weisshaar B., Martin C., Lepiniec L. (2010). MYB transcription factors in Arabidopsis. Trends Plant Sci..

[125] Stracke R., Ishihara H., Huep G., Barsch A., Mehrtens F., Niehaus K., Weisshaar B. (2007). Differential regulation of closely related R2R3-MYB transcription factors controls flavonol accumulation in different parts of the Arabidopsis thaliana seedling. Plant J..

[126] Borevitz J.O., Xia Y., Blount J., Dixon R.A., Lamb C. (2000). Activation tagging identifies a conserved MYB regulator of phenylpropanoid biosynthesis. Plant Cell.

[127] Matsui K., Umemura Y., Ohme-Takagi M. (2008). AtMYBL2, a protein with a single MYB domain, acts as a negative regulator of anthocyanin biosynthesis in Arabidopsis. Plant J..

[128] Quattrocchio F., Verweij W., Kroon A., Spelt C., Mol J., Koes R. (2006). PH4 of Petunia is an R2R3 MYB protein that activates vacuolar acidification through interactions with basic-helix-loop-helix transcription factors of the anthocyanin pathway. Plant Cell.

[129] Quattrocchio F., Wing J., van der Woude K., Souer E., de Vetten N., Mol J., Koes R. (1999). Molecular analysis of the anthocyanin2 gene of petunia and its role in the evolution of flower color. Plant Cell.

[130] Kobayashi S., Yamamoto N.G., Hirochika H. (2005). Association of VvmybA1 gene expression with anthocyanin production in grape (Vitis vinifera) skin-color mutants. J. Jpn. Soc. Hortic. Sci..

[131] Kobayashi S., Goto-Yamamoto N., Hirochika H. (2004). Retrotransposon-induced mutations in grape skin color. Science.

[132] Deluc L., Barrieu F., Marchive C., Lauvergeat V., Decendit A., Richard T., Carde J.P., Merillon J.M., Hamdi S. (2006). Characterization of a grapevine R2R3-MYB transcription factor that regulates the phenylpropanoid pathway. Plant Physiol..

[133] Czemmel S., Stracke R., Weisshaar B., Cordon N., Harris N.N., Walker A.R., Robinson S.P., Bogs J. (2009). The grapevine R2R3-MYB transcription factor VvMYBF1 regulates flavonol synthesis in developing grape berries. Plant Physiol..

[134] Mano H., Ogasawara F., Sato K., Higo H., Minobe Y. (2007). Isolation of a regulatory gene of anthocyanin biosynthesis in tuberous roots of purple-fleshed sweet potato. Plant Physiol..

[135] Espley R.V., Hellens R.P., Putterill J., Stevenson D.E., Kutty-Amma S., Allan A.C. (2007). Red colouration in apple fruit is due to the activity of the MYB transcription factor, MdMYB10. Plant J..

[136] Ban Y., Honda C., Hatsuyama Y., Igarashi M., Bessho H., Moriguchi T. (2007). Isolation and functional analysis of a MYB transcription factor gene that is a key regulator for the development of red coloration in apple skin. Plant Cell Physiol..

[137] Peel G.J., Pang Y., Modolo L.V., Dixon R.A. (2009). The LAP1 MYB transcription factor orchestrates anthocyanidin biosynthesis and glycosylation in Medicago. Plant J..

[138] Akagi T., Ikegami A., Tsujimoto T., Kobayashi S., Sato A., Kono A., Yonemori K. (2009). DkMyb4 is a Myb transcription factor involved in proanthocyanidin biosynthesis in persimmon fruit. Plant Physiol..

[139] Hou D.X., Kumamoto T. (2010). Flavonoids as protein kinase inhibitors for cancer chemoprevention: Direct binding and molecular modeling. Antioxid. Redox Signal..

[140] Muday G.K., Murphy A.S. (2002). An emerging model of auxin transport regulation. Plant Cell.

[141] Peer W.A., Murphy A.S. (2007). Flavonoids and auxin transport: Modulators or regulators?. Trends Plant Sci..

[142] Jacobs M., Rubery P.H. (1988). Naturally occurring auxin transport regulators. Science.

[143] Brown D.E., Rashotte A.M., Murphy A.S., Normanly J., Tague B.W., Peer W.A., Taiz L., Muday G.K. (2001). Flavonoids act as negative regulators of auxin transport *in vivo* in arabidopsis. Plant Physiol..

[144] Buer C.S., Muday G.K. (2004). The transparent testa4 mutation prevents flavonoid synthesis and alters auxin transport and the response of Arabidopsis roots to gravity and light. Plant Cell.

[145] Buer C.S., Djordjevic M.A. (2009). Architectural phenotypes in the transparent testa mutants of Arabidopsis thaliana. J. Exp. Bot..

[146] Lewis D.R., Miller N.D., Splitt B.L., Wu G., Spalding E.P. (2007). Separating the roles of acropetal and basipetal auxin transport on gravitropism with mutations in two Arabidopsis multidrug resistance-like ABC transporter genes. Plant Cell.

[147] Murphy A.S., Hoogner K.R., Peer W.A., Taiz L. (2002). Identification, purification, and molecular cloning of N-1-naphthylphthalmic acid-binding plasma membrane-associated aminopeptidases from Arabidopsis. Plant Physiol..

[148] Noh B., Murphy A.S., Spalding E.P. (2001). Multidrug resistance-like genes of Arabidopsis required for auxin transport and auxin-mediated development. Plant Cell.

[149] Friml J., Benkova E., Blilou I., Wisniewska J., Hamann T., Ljung K., Woody S., Sandberg G., Scheres B., Jurgens G. (2002). AtPIN4 mediates sink-driven auxin gradients and root patterning in Arabidopsis. Cell.

[150] Friml J., Wisniewska J., Benkova E., Mendgen K., Palme K. (2002). Lateral relocation of auxin efflux regulator PIN3 mediates tropism in Arabidopsis. Nature.

[151] Galweiler L., Guan C., Muller A., Wisman E., Mendgen K., Yephremov A., Palme K. (1998). Regulation of polar auxin transport by AtPIN1 in Arabidopsis vascular tissue. Science.

[152] Muller A., Guan C., Galweiler L., Tanzler P., Huijser P., Marchant A., Parry G., Bennett M., Wisman E., Palme K. (1998). AtPIN2 defines a locus of Arabidopsis for root gravitropism control. EMBO J..

[153] Hernandez I., Alegre L., van Breusegem F., Munne-Bosch S. (2009). How relevant are flavonoids as antioxidants in plants?. Trends Plant Sci..

[154] Lewis D.R., Negi S., Sukumar P., Muday G.K. (2011). Ethylene inhibits lateral root development, increases IAA transport and expression of PIN3 and PIN7 auxin efflux carriers. Development.

[155] DeLong A., Mockaitis K., Christensen S. (2002). Protein phosphorylation in the delivery of and response to auxin signals. Plant Mol. Biol..

[156] Benjamins R., Quint A., Weijers D., Hooykaas P., Offringa R. (2001). The PINOID protein kinase regulates organ development in Arabidopsis by enhancing polar auxin transport. Development.

[157] Galvan-Ampudia C.S., Offringa R. (2007). Plant evolution: AGC kinases tell the auxin tale. Trends Plant Sci..

[158] Friml J., Yang X., Michniewicz M., Weijers D., Quint A., Tietz O., Benjamins R., Ouwerkerk P.B., Ljung K., Sandberg G. (2004). A PINOID-dependent binary switch in apical-basal PIN polar targeting directs auxin efflux. Science.

[159] Zourelidou M., Absmanner B., Weller B., Barbosa I.C., Willige B.C., Fastner A., Streit V., Port S.A., Colcombet J., de la Fuente van Bentem S. (2014). Auxin efflux by PIN-FORMED proteins is activated by two different protein kinases, D6 PROTEIN KINASE and PINOID. eLife.

[160] Zegzouti H., Anthony R.G., Jahchan N., Bogre L., Christensen S.K. (2006). Phosphorylation and activation of PINOID by the phospholipid signaling kinase 3-phosphoinositide-dependent protein kinase 1 (PDK1) in Arabidopsis. Proc. Natl. Acad. Sci. USA.

[161] Geisler M., Blakeslee J.J., Bouchard R., Lee O.R., Vincenzetti V., Bandyopadhyay A., Titapiwatanakun B., Peer W.A., Bailly A., Richards E.L. (2005). Cellular efflux of auxin catalyzed by the Arabidopsis MDR/PGP transporter AtPGP1. Plant J..

[162] Geisler M., Murphy A.S. (2006). The ABC of auxin transport: The role of p-glycoproteins in plant development. FEBS Lett..

[163] Kanneganti V., Gupta A.K. (2008). Wall associated kinases from plants—An overview. Physiol. Mol. Biol. Plants.

[164] Potters G., Pasternak T.P., Guisez Y., Palme K.J., Jansen M.A. (2007). Stress-induced morphogenic responses: Growing out of trouble?. Trends Plant Sci..

[165] Potters G., Pasternak T.P., Guisez Y., Jansen M.A. (2009). Different stresses, similar morphogenic responses: Integrating a plethora of pathways. Plant Cell Environ..

[166] Mathesius U. (2001). Flavonoids induced in cells undergoing nodule organogenesis in white clover are regulators of auxin breakdown by peroxidase. J. Exp. Bot..

[167] Jansen M.A., van den Noort R.E., Tan M.Y., Prinsen E., Lagrimini L.M., Thorneley R.N. (2001). Phenol-oxidizing peroxidases contribute to the protection of plants from ultraviolet radiation stress. Plant Physiol..

[168] Sugiyama A., Shitan N., Yazaki K. (2007). Involvement of a soybean ATP-binding cassette-type transporter in the secretion of genistein, a signal flavonoid in legume-Rhizobium symbiosis. Plant Physiol..

[169] Badri D.V., Loyola-Vargas V.M., Broeckling C.D., de-la-Pena C., Jasinski M., Santelia D., Martinoia E., Sumner L.W., Banta L.M., Stermitz F. (2008). Altered profile of secondary metabolites in the root exudates of Arabidopsis ATP-binding cassette transporter mutants. Plant Physiol..

[170] Masaoka Y., Kojima M., Sugihara S., Yoshihara T., Koshino M., Ichihara A., Koshino M., Ichihara A. (1993). Dissolution of ferric phosphates by alfalfa (*Medicago sativa* L.) root exudates. Plant Soil.

[171] Cesco S., Neumann G., Tomasi N., Pinton R., Weisskopf L. (2010). Release of plant-borne flavonoids into the rhizosphere and their role in plant nutrition. Plant Soil.

[172] Johnson E.T., Berhow M.A., Dowd P.F. (2007). Expression of a maize Myb transcription factor driven by a putative silk-specific promoter significantly enhances resistance to Helicoverpa zea in transgenic maize. J. Agric. Food Chem..

[173] Yan Z., Guo H., Yang J., Liu Q., Jin H., Xu R., Cui H., Qin B. (2014). Phytotoxic flavonoids from roots of *Stellera chamaejasme* L. (Thymelaeaceae). Phytochemistry.

[174] Tanaka Y., Brugliera F., Chandler S. (2009). Recent progress of flower colour modification by biotechnology. Int. J. Mol. Sci..

[175] Tanaka Y., Brugliera F., Kalc G., Senior M., Dyson B., Nakamura N., Katsumoto Y., Chandler S. (2010). Flower color modification by engineering of the flavonoid biosynthetic pathway: Practical perspectives. Biosci. Biotechnol. Biochem..

[176] Nishihara M., Nakatsuka T. (2011). Genetic engineering of flavonoid pigments to modify flower color in floricultural plants. Biotechnol. Lett..

[177] Saleem M., Nazir M., Ali M.S., Hussain H., Lee Y.S., Riaz N., Jabbar A. (2010). Antimicrobial natural products: An update on future antibiotic drug candidates. Nat. Prod. Rep..

[178] Russo G.L., Russo M., Spagnuolo C. (2014). The pleiotropic flavonoid quercetin: From its metabolism to the inhibition of protein kinases in chronic lymphocytic leukemia. Food Funct..

[179] Middleton E., Kandaswami C., Theoharides T.C. (2000). The effects of plant flavonoids on mammalian cells: Implications for inflammation, heart disease, and cancer. Pharmacol. Rev..

[180] Cho B.O., So Y., Jin C.H., Nam B.M., Yee S.T., Jeong I.Y. (2014). 3-Deoxysilybin exerts anti-inflammatory effects by suppressing NF-kappaB activation in lipopolysaccharide-stimulated RAW264.7 macrophages. Biosci. Biotechnol. Biochem..

[181] Cheng D., Zhang Y., Gao D., Zhang H. (2014). Antibacterial and anti-inflammatory activities of extract and fractions from *Pyrrosia petiolosa* (Christ et Bar.) Ching. J. Ethnopharmacol..

[182] Kritas S.K., Saggini A., Varvara G., Murmura G., Caraffa A., Antinolfi P., Toniato E., Pantalone A., Neri G., Frydas S. (2013). Luteolin inhibits mast cell-mediated allergic inflammation. J. Biol. Regul. Homeost. Agents.

[183] Lee W., Ku S.K., Bae J.S. (2014). Antiplatelet, anticoagulant, and profibrinolytic activities of baicalin. Arch. Pharm. Res..

[184] Gaur R., Yadav K.S., Verma R.K., Yadav N.P., Bhakuni R.S. (2014). *In vivo* anti-diabetic activity of derivatives of isoliquiritigenin and liquiritigenin. Phytomedicine.

[185] Salvamani S., Gunasekaran B., Shaharuddin N.A. (2014). Antiartherosclerotic effects of plant flavonoids. BioMed Res. Int..

[186] Bogdan Allemann I., Baumann L. (2008). Antioxidants used in skin care formulations. Skin Ther. Lett..

[187] Skorkowska-Telichowska K., Zuk M., Kulma A., Bugajska-Prusak A., Ratajczak K., Gasiorowski K., Kostyn K., Szopa J. (2010). New dressing materials derived from transgenic flax products to treat long-standing venous ulcers—A pilot study. Wound Repair Regen..

[188] Saslowsky D.E., Warek U., Winkel B.S. (2005). Nuclear localization of flavonoid enzymes in Arabidopsis. J. Biol. Chem..

[189] Naoumkina M., Dixon R.A. (2008). Subcellular localization of flavonoid natural products. Plant Signal. Behav..

